# Clinical outcomes of postoperative extracorporeal membrane oxygenation support in Stanford type a aortic dissection

**DOI:** 10.1186/s12871-021-01252-6

**Published:** 2021-02-05

**Authors:** Fudong Fan, Qing Zhou, Jun Pan, Hailong Cao, Kai Li, Yunxing Xue, Min Ge, Xuan Luo, Yang Chen, Dongjin Wang

**Affiliations:** Department of Cardiothoracic Surgery, Nanjing Drum Tower Hospital, The Affiliated Hospital of Nanjing University Medical School, 210008 Nanjing, China

**Keywords:** Stanford type a aortic dissection, Extracorporeal membrane oxygenation support, Clinical outcome

## Abstract

**Background:**

Extracorporeal membrane oxygenation (ECMO) support may be considered to reduce mortality but survival and clinical outcomes are uncertain after Stanford type A Aortic dissection (TAAD). We analyzed the data of TAAD patients with postoperative ECMO support in our institution to investigate clinical outcomes.

**Methods:**

In this retrospective cohort study, all clinical data of TAAD patients with postoperative ECMO support from January 2013 to October 2019 in our institution were harvested. Cases with redo or incomplete records were excluded.

**Results:**

22 cases were enrolled, 18 male and 4 female. The mean age was52.85±10.91 years. 20 patients underwent VA-ECMO treatment and 2 patients received VV-ECMO support. The support time was92.54±78.71 hours. 9 patients were successfully weaned from ECMO. 30-day in-hospital survival rate was 27.27 % (6/22). The follow-up duration is from 5 to 74 months. The median follow-up time is 35 months. Only four patients were still alive at the end of the follow-up period.

**Conclusions:**

The mortality of TAAD patients with postoperativesevere circulatory and respiratory dysfunctions is high. ECMO would be considered as a valuable contribution to save lives. But more experience needs to be accumulated to improve clinical outcome.

## Background

Stanford type A aortic dissection (TAAD) is a life-threatening aortic emergency with high morbidity and mortality. Without any management, more than 50 % patients will be dead from aortic rupture or organ malperfusion in the first two days after onset [1]. At present, surgery is considered to be the most effective treatment to save life. Based on the results from the International Registry of Acute Aortic Dissection (IRAD), the rate of TAAD patients undergoing surgery had increased to 90 % in the past two decades and a growing number of patients were survival by surgical treatment [1]. However, although free of the death from acute aortic rupture, some patients still needed to struggle with postoperative complications. Due to critical preoperative condition and longer operation time, postoperative circulatory and respiratory dysfunction were more usual than conventional cardiac surgery. Almost every patient needed to undergo deep hypothermia circulatory arrest during the aortic repair which also increased the risk of perioperative management. Several frontline support methods such as mechanical ventilation and vasoactive drugs were frequently deployed, but could not be helpful for all patients. Intra-aortic balloon pump (IABP) via the femoral artery often applied for patients receiving conventional cardiac surgery do not allowed to be placed for TAAD patients because of residue dissection in descending aorta. Mechanical circulatory and respiratory support may function efficiently. Extracorporeal membrane oxygenation (ECMO) support was originated from cardiopulmonary bypass technique which had been widely used for severe circulatory and/or respiratory dysfunction. With the help of ECMO therapy, many victims suffering from myocardial infarction, pulmonary embolism and acute lung injury could obtain the chance to recover [2–4]. However, there were little reports concentrating on ECMO support in TAAD patients and the clinical outcome of these subjects was unclear. Here we analyzed the data of TAAD patients with postoperative ECMO support in our institution to investigate survival and clinical outcomes.

## Methods

### Patients selection

This is a retrospective cohort study. From January 2013 to October 2019, all clinical data of TAAD patients at the Department of Cardiothoracic Surgery in Nanjing Drum Tower Hospital of Nanjing University medical school were collected. In all patients, the aortic dissection was diagnosed with the aid of either computerized tomography or magnetic resonance imaging before surgical treatment. Subjects with reoperation or incomplete clinical records were ruled out. Clinical variables including basic characteristics, perioperative data, in-hospital and follow-up outcome. In all the enrolled TAAD patients, only 22 patients underwent ECMO support after operation, so these 22 patients were include in the current study. Our work was approved by the Ethics committee of Nanjing Drum Tower Hospital (Approval number 2020-281-01), and all subjects gave research authorization.

### Operative technique

Almost all of patients received operation immediately after admission. Cardiopulmonary bypass was established through femoral and/or axillary artery and right atrium. Single selective cerebral perfusion via axillary artery was used for cerebral protection under deep hypothermia circulatory arrest at the nasal temperature of about 20 to 22 degrees Celsius when aortic arch needed to be repair. For patients with elderly age or critical preoperative condition, ascending aorta replacement combined with hemiarch replacement had been chosen usually under sole circulatory arrest without cerebral perfusion at the nasal temperature of about 25 degrees Celsius. If primary intimal tear located at aortic arch, we often replaced total arch and deployed frozen elephant trunk in descending aorta. Aortic root reconstruction technique (Sandwich reinforcement methods with artificial graft and dacron patch) was used for cases with aortic sinus involved. We also did valve repair or replacement if patients had aortic valve function impairment. When patients were concomitant with myocardial ischemia, coronary arteries bypass grafting was considered to perform. However, final surgical strategy was made by surgeons according to intraoperative findings.

### ECMO support

**Fig. 1:**
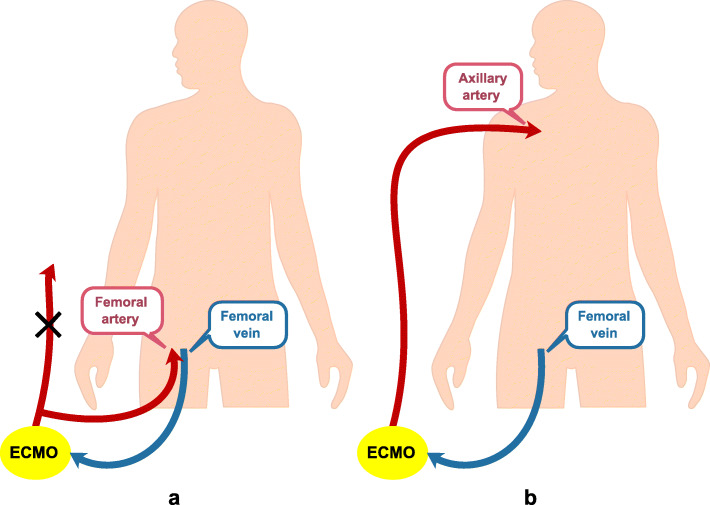
The procedure of ECMO for emergen**cy** cases a Two arterial conduits were prepared in advance and VA-ECMO treatment was performed via femoral artery (connected with one of the two arterial conduits) and vein firstly. **b** After hemodynamic condition was stable, another arterial conduit was connected with axillary artery and then previous arterial cannulas through femoral artery was removed

After operation, all patients were transferred to intensive care unit. When patients presented circulatory and/or respiratory failure with escalation of vasoactive agents and/or mechanical ventilation support, we would quickly evaluate the condition and make sure if ECMO therapy should be established. For cases who were failure to wean from cardiopulmonary bypass after procedure, we used ECMO support immediately in the operation room. The indication of ECMO use in our institution included (1) persistent hypotension with an inotropic equivalent score exceeding 50 (defined as mg/kg/min = dopamine + dobutamine + epinephrine×100 + norepinephrine×100 + isoproterenol×100 + milrinone × 15); (2) sustained hypoxemia with oxygenation index < 100 mmHg; (3) reversible organ dysfunction; (4) no active bleeding. The ECMO circuit consisted of a centrifugal pump (Rotaflow; Maquet Getinge Group, Germany) with a suit of conduits and microporous membrane oxygenator (Hilite BE-PLS 2050; Maquet Getinge Group, Germany). Previously we placed cannulas through femoral artery and vein to run VA-ECMO. Because sometimes we could not determine the location of intimal tear and hemodynamic condition in false lumen in residue dissected aorta, the femoral-femoral bypass would frequently lead to upper body ischemia. Recently we preferred to perform VA-ECMO support through axillary artery and femoral vein which might provide the antegrade perfusion to avoid cerebral ischemia. If adequate time was acquired to prepare for ECMO establishment, a short 8mm graft would be anastomosed to native artery as to avoid distal limb ischemia. For cases requiring emergency ECMO therapy, we often prepared two arterial conduits in advance and quickly gave VA-ECMO treatment via femoral artery (connected with one of the two arterial conduits) and vein firstly. After hemodynamic condition was stable, another arterial conduit was connected with axillary artery using a short 8mm graft and then previous arterial cannulas through femoral artery was removed (Showed in Fig. 1). We performed VV-ECMO support with cannulas through jugular vein and femoral vein. We gave High blood flow (about 80-100ml/kg) to improve arterial oxygen levels rapidly. However, for patients supported by VA-ECMO, if end-organs perfusion was adequate, we would like to provide half blood flow (about 40-60ml/kg) rather than higher blood flow (60-80ml/kg usually). It had been found that proper cardiac preload existence seemed to be helpful for heart function recovery in our practice. Heparin and protamine were administrated intravenously to maintain activated clotting time (ACT) between 180 and 200 seconds. Circulatory and respiratory function monitor was implemented as usual. During ECMO support, mean arterial pressure (MAP), heart rate (HR), respiratory rate (RR), mechanical ventilation parameters, arterial blood gas values, partial pressure of oxygen (PaO_2_), partial pressure of carbon dioxide (PaCO_2_), pondus hydrogenii (PH), arterial oxygen saturation, serum lactate levels were followed. Serial measurement of hemoglobin and platelet, urine output, and renal and hepatic function were also taken. The dosage of inotropes were regulated according to the hemodynamic condition and echocardiography. For patients with ECMO support, we performed arterial blood gas test once an hour. During the proceeding of ECMO therapy, all subjects were given mechanical ventilation and sedated with midazolam and fentanyl infusion. The parameters of ventilation were modulated depending on arterial blood gas values. Albumin solution and blood plasma were often used to provide effective circulating blood volume and reduce edema. For most patients we often prescribed vancomycin and third-generation cephalosporin until ECMO weaning. Once bowel sound returned, enteral nutrition was applied by the way of nasoenteral tube.

### Weaning from ECMO

We initiated weaning when patients were in stable hemodynamic condition (CVP ≤ 15 mmHg, MAP ≥ 65 mmHg, pulse pressure ≥ 30 mmHg) supported by low doses of vasoactive drugs. Echocardiography was applied to evaluate heart function (left ventricle eject fraction ≥ 30 %, no right and left ventricle distention, aortic velocity-time integral > 10 cm). Respiratory function should be fine aided by mechanical ventilation with acceptable variables before weaning. We gradually reduced blood flow to less than 1.0L/min and then considered to wean off. The ECMO cannulas were removed and wound was closed in the operation room. Conventional management was given as the other cardiovascular surgical patients after weaning.

### Data analysis

We recorded variables including patient age, gender, preoperative presentation, operative procedures, cardiopulmonary bypass time, ECMO weaning rate and clinical outcome including in-hospital survival and follow-up information. Continuous, parametric variables were presented as means ± standard deviation and categorical variables as percentage. Analyses were performed using SPSS 19.0 (IBM, Armonk, NY).

## Results

22 patients were enrolled finally from 1108 consecutive TAAD cases with eighteen male and four female. The mean age was 52.85 ± 10.91 years. 17 patients (77.27 %) had the history of hypertension. No person was combined with connective tissue diseases or hereditary diseases. The average body mass index (BMI) was 24.75 ± 4.13 kg/m^2^. Preoperative cardiac tamponade presented in 7 cases (31.82 %). 13 patients were concomitant with one or more organs malperfusion. The characteristics of patients was showed in Table 1.

**Table 1:** The preoperative characteristics of patients

Variables	*N* = 22
Gender (male, n, %)	18 (81.82 %)
Age (years, mean ± SD)	52.85 ± 10.91
Body mass index (kg/m^2^, mean ± SD)	24.75 ± 4.13
Hypertension (n, %)	17 (77.27 %)
Connective tissue diseases (n, %)	0
Hereditary diseases (n, %)	0
Smoking (n, %)	11 (50.00 %)
Cardiac tamponade (n, %)	7 (31.82 %)
Coronary arteries ischemia (n, %)	7 (31.82 %)
Cerebral ischemia (n, %)	1 (4.55 %)
Renal ischemia (n, %)	4 (18.18 %)
Intestinal ischemia (n, %)	1 (4.55 %)
Limb ischemia (n, %)	3 (13.64 %)

All patients underwent emergency operation under general anaesthesia and cardiopulmonary bypass. In seven patients, ascending aorta and hemi-arch replacement was performed lonely. Total arch replacement and frozen elephant trunk procedure were operated in fifteen cases. In seven patients, aortic valve replacement was performed. Nineteen patients underwent aortic root reconstruction. In addition, coronary arteries bypass grafting (CABG) was employed for 5 patients because of coronary artery ostia involved. Intraoperative details were summarized in Table 2.

**Table 2:** Intraoperative information

Variables	*N* = 22
Ascending aorta and hemi-arch replacement (n, %)	7 (27.27 %)
Total arch replacement and frozen elephant trunk procedure (n, %)	15 (77.27 %)
Aortic valve replacement (n, %)	7 (27.27 %)
Aortic root reconstruction (n, %)	19 (86.36 %)
Coronary arteries bypass grafting (n, %)	5 (22.73 %)
Operation time (min, mean ± SD)	491.32 ± 123.56
Cardiopulmonary bypass time (min, mean ± SD)	375.44 ± 110.17
Aortic cross clamp time (min, mean ± SD)	237.85 ± 106.94
Deep hypothermia circulatory arrest time (min, mean ± SD)	31.09 ± 15.03

**Fig. 2:**
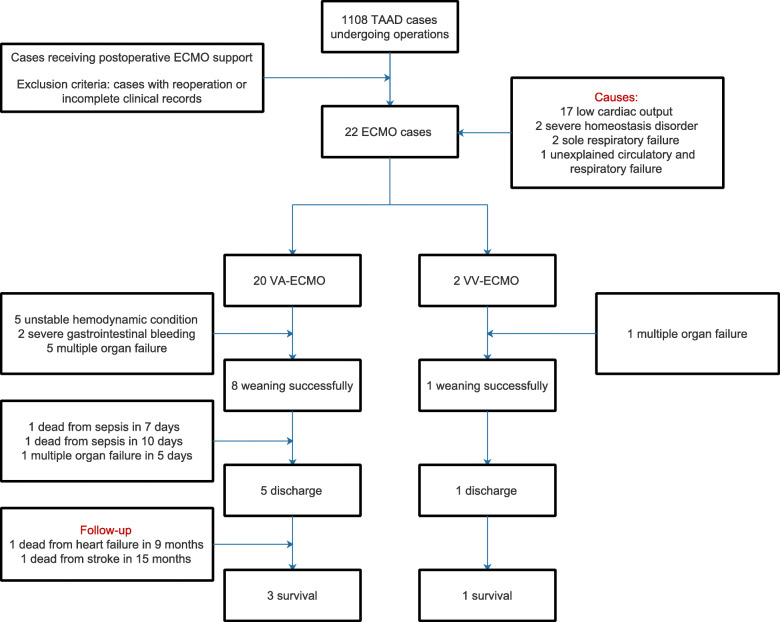
The enrollment, allocation, follow-up, and analysis of TAAD patients from January 2013 to October 2019 in our institution

20 patients were supported by VA-ECMO and 2 patients by VV-ECMO therapy. The causes of ECMO support included postoperative low cardiac output in 17 patients, severe homeostasis disorder in 2, sole respiratory failure in 2 and unexplained circulatory and respiratory failure in 1. The causes of ECMO support included postoperative low cardiac output in 17 patients, severe homeostasis disorder in 2, sole respiratory failure in 2 and unexplained circulatory and respiratory failure in 1.The mean support time was 92.54 ± 78.71 hours and intensive care unit stay was 7.91 ± 10.89 days. 9 patients were successfully weaned from ECMO (1 from VV-ECMO and 8 from VA-ECMO). In 6 successful weaning patients with VA-ECMO, arterial cannulas were placed through axillary artery. From a total of 22 patients, three patients died within 10 days. The pathophysiologic background of death was considered as: two patients died from sepsis after ECMO weaning on day 7 and day 10, in one patient multiple organ failure was considered on day 5 after ECMO weaning. The in-hospital survival rate was 27.27 % (6/22). The mean ICU stay was 7.91 ± 10.89 days and the mean follow-up time in the ward was 10.24 ± 11.57 days. Patients were permitted to discharge when they had stable circulatory and respiratory condition assisted by oral medicine, satisfied lab test value (such as hemoglobin value more than 9.0 g/dl and normal coagulation) and imaging examination results and no complains. The follow-up duration is from 5 to 74 months. The median follow-up time was 35 months. All patients were followed up with history harvesting, physical examination, lab test, electrocardiogram, echocardiography and CT scanning in the clinic. The follow-up data showed only 4 patients still alive. The duration of follow-up differed for each patients and the follow-up durations are as follows for each patient: 5 months, 55 months, 61 months and 74 months respectively. There were one patient with the NYHA Class II and three patients with Class III during follow-up. At the end of follow-up period, one patient had lower limb neurological dysfunction with bed-ridden and the other three patients had normal neurological function. One patient need persistent dialysis because of unrecoverable renal dysfunction. The enrollment, allocation, follow-up, and analysis of TAAD patients were shown in Fig. 2. During the follow-up period, there was only one patient undergoing bioprosthetic replacement who need to take warfarin under the monitor of prothrombin time for six months. The other patients should keep normal coagulation. The clinical outcome summary and follow-up information was listed in Tables 3 and 4 respectively.

**Table 3:** ECMO support details and clinical outcomes

Variables	*N* = 22
VA-ECMO (n, %)	20 (90.91 %)
VV-ECMO (n, %)	2 (9.09 %)
Causes of ECMO support	
Postoperative low cardiac output (n, %)	17 (77.27 %)
Severe homeostasis disorder (n, %)	2 (9.09 %)
Sole respiratory failure (n, %)	2 (9.09 %)
Unexplained circulatory and respiratory failure (n, %)	1 (4.55 %)
ECMO support time (hours, mean ± SD)	92.54 ± 78.71
Intensive care unit stay (days, mean ± SD)	7.91 ± 10.89
Weaned from ECMO successfully (n, %)	9 (40.91 %)
In-hospital survival (n, %)	6 (27.27 %)
Renal failure	17(77.27 %)
Neurological dysfunction	8(36.36 %)

**Table 4:** Follow-up information after discharge

Variables	*N* = 6	*N* = 4 (survival)
Long-term survival (n, %)	4 (66.67 %)	-
Bed-ridden status (n, %)	1 (16.67 %)	1(25.00 %)
Heart function		
*NYHA Class II*	2(33.33 %)	1(25.00 %)
*NYHA Class III*	4(66.67 %)	3(75.00 %)
Respiratory dysfunction	2 (33.33 %)	0(0.00 %)
Renal dysfunction	1 (16.67 %)	1(25.00 %)
Liver dysfunction	0 (0.00 %)	0(0.00 %)
Neurological dysfunction	2(33.33 %)	1(25.00 %)
Stroke	1 (16.67 %)	0(0.00 %)
Gastrointestinal bleeding	1 (16.67 %)	0(0.00 %)
Antiarrhythmic drugs use	2 (33.33 %)	1(25.00 %)
Warfarin use	1 (16.67 %)	1(25.00 %)
Diuretic use	2 (33.33 %)	1(25.00 %)
Antihypertensive agents use	5 (83.33 %)	3(75.00 %)

## Discussion

TAAD is a fatal cardiovascular emergency with high morbidity and mortality. Surgical treatment is the most effective method to substantially reduce mortality for these patients. Because of the differences in intimal tear location, hemodynamic condition in false and true lumen and involving extent of aorta among TAAD patients, individual perioperative treatment strategy needs to be adopted [1]. When patients are concomitant with organ malperfusion or cardiac tamponade after onset, postoperative circulatory and/or respiratory dysfunction are more common and prognosis could be even worse. Common treatment may not be always working for these patients. Sustained hypotension and hypoxemia are life-threatening which would lead to patient death in a much short time. Temporary circulatory and respiratory function support methods may be useful for winning time to save lives. ECMO support has been widely employed in the treatment of heart and lung failure caused by many kinds of disease [2, 3]. It has been showed that the clinical outcomes of patients with ECMO use in the setting of other diseases such as fulminant myocarditis and pulmonary embolism were really acceptable [4]. For patients with post-cardiotomy refractory cardiogenic shock, the survival rate could be higher than 50 % [5]. However, until to now, no protocol for the indication and practice of ECMO support in TAAD patients can be obtained. In this paper, we retrospectively collected the clinical data in our institution to investigate the survival and prognosis of these patients.

There were few articles focusing on outcome of TAAD patients supported by ECMO. Lin and his colleagues reported the short-term and long-term outcome of 20 cases with VA-ECMO support from 162 consecutive TAAD patients at a medical center of Taiwan. All patients presented post-cardiotomy cardiogenic shock. The arterial cannulas was placed through right axillary or femoral artery and the venous cannulas was obtained from right atrial (delayed sternal wound closure) or femoral vein (sternal wound closure). However, as same as our experience, it had been thought that using the femoral artery for ECMO access may not be a good choice in TAAD patients because of the residual dissection and intimal tear in the remnant aorta. Finally, there were 13 cases weaning off successfully in the group but only 7 patients alive. The in-hospital survival rate was 35 %. Causes of death included sepsis, bowel ischemia and massive bleeding associated with aortoesophageal fistula. The follow-up data suggested the postdischarge survival rate was equivalent in patients with or without ECMO support. For the patients having long-term survival, cardiac function returned to almost normal [6]. An another observational study from the United States enrolled the most number of TAAD patients assisted by ECMO so far. Clinical data was extracted from The Pennsylvania Health Care Cost Containment Council (PHC4) database. A total of 35 patients underwent TAAD repair and VA-ECMO treatment. Of them, 31 patients underwent open surgery and 4 patients received interventional procedure. ECMO was instituted on the same day of TAAD surgery in most of patients (69.2 %). Median time from ECMO deployment to death was about one day. The prognosis for these subjects was much poor and only 10.3 % of patients were still survival in discharge. The higher mortality was considered to be associated with persistent acidosis caused by visceral or lower extremity malperfusion although undergoing aortic repair [7]. Recently, there was a clinical report showing better outcome in a medical center of Northern China. Only 7 cases had been included from consecutive 246 acute TAAD victims. The mean support time was 244.5 ± 57.8 hours. Consistent with our practice, a short 8mm graft had been also anastomosed to native artery in order to avoid limb ischemia. It was believed that the early extubation strategy could improve diagnosis. So most of patients had been removed intubation before weaning off. All of 7 patients were weaned from ECMO successfully. Only one person was dead from cardiac arrest after weaning. The other six patients survived to discharge who were still alive during the median follow-up of 19 months [8]. However, the enrolled patients requiring ECMO support in this report were all caused by coronary arteries involved or poor myocardial protection. With the help of ECMO therapy, adequate time was achieved to wait for heart function recovery.

Our results indicated the worse outcome of patients with ECMO use in the setting of TAAD and the 30-days in-hospital survival rate of these cases in our institution was less than 30 %. In fact, the poor outcome was also found in the reports from Taiwan and the United States. It was thought that various preoperative condition might be relative to high mortality. Unlike most patients with valvular heart disease or coronary heart disease, many TAAD patients were concomitant with critical status such as cardiac tamponade or severe organ malperfusion which often increased the risk of perioperative treatment. Because the location of intimal tear in residue dissected aorta and hemodynamic condition in false lumen could not be understood completely during the perioperative management, there was potential risk of cerebral, abdominal organs and lower limbs ischemia in the processing of VA-ECMO therapy. Retrograde blood inflow from cannulas through femoral artery could lead to false lumen dilation and aggravation of visceral and cerebral ischemia. Now we preferred to place arterial cannulas through axillary artery to provide antegrade perfusion. For cases requiring emergency VA-ECMO support, we would like to prepare two arterial conduits in advance and establish VA-ECMO treatment via femoral artery (connected with one of the two arterial conduits) and vein firstly. After hemodynamic condition was stable, another arterial conduit was connected with axillary artery using a short 8mm graft. Then the first arterial conduit was clamped and previous arterial cannulas through femoral artery was removed. Antegrade perfusion could reduce the likelihood of false lumen dilation in remnant aorta and avoid cerebral and visceral ischemia. Our experience suggested this method seemed to decrease the incidence of complication and improve the diagnosis of these patients. In any case, some problems such as myocardial cell necrosis, intestinal ischemia and severe homeostasis disorder could not be resolved by surgical treatment and ECMO use. These problems still exist and influence on the survival rate of TAAD victims.

During the VA-ECMO, if end-organs perfusion was adequate, we preferred to provide lower blood flow (about 40-60ml/kg) rather than higher blood flow (60-80ml/kg usually). We found that proper cardiac preload existence might be helpful for heart function recovery. With the support of ECMO, we gradually reduced the dosage of inotropes to avoid the side effects. On the contrary to the management reported by Wang and his colleagues, we would like to keep mechanical ventilation under sedation which could decrease tissue oxygen consumption. Serum lactate levels had been considered to have relationship with the prognosis of patients. Higher serum lactate levels often suggested shock or visceral ischemia. This condition needed to be corrected as soon as possible. The parameter of ventilation, dosage of inotropes or blood flow of ECMO could be modulated. During ECMO support, it was necessary for patients to receive blood transfusion according to the result of lab test. After transferring to inward, patients were under the monitor of lab test, electrocardiogram and echocardiography. Rehabilitation training was also important for patients weaning from ECMO to improve the quality of their lives. In agreement with Lin and his colleagues’ report, it have been found in our follow-up information that most of the survival patients had normal heart function. We would proceed to perform follow-up for these patients in the clinic in order to provide more valuable information.

Nevertheless, the population of TAAD patients who need to be supported by ECMO remained relatively low and there was inevitable selection bias in our work. Because few clinical reports concentrated on outcome of TAAD patients with ECMO support, more experiences needed to be obtained. Present report could not provide a mature protocol for indication, timing and clinical practice of ECMO use in the setting of TAAD. In the future, multivariate regression and propsensity matching analysis may supply more information about who had the higher risk of receiving ECMO support after aortic repair. This is now just a single center study, which is also the limitation for this study. We will consider multicenter controlled research and provide more evidence to support our data.

## Conclusions

The mortality of TAAD patients with postoperative ECMO support was still high. But when TAAD patients are combined with refractory postoperative circulatory and respiratory dysfunction, ECMO support is still reasonable to achieve the mortality reduction. Proper surgical strategy and careful management of perioperative complications may increase the survival rate of these patients. But more experiences need to be accumulated to improve clinical outcome.

## Data Availability

The datasets used and/or analyzed during the current study are available from the corresponding author on reasonable request. FF and XX had full access to all of the data in the study and take responsibility for the integrity of the data and the accuracy of the data analysis.

## Abbreviations

TAAD: Stanford Type A aortic dissection
ECMO: Extracorporeal membrane oxygenation
CT: Computed Tomography
VA-ECMO: Veno-arterial extracorporeal membrane oxygenation
VV-ECMO: Veno-venous extracorporeal membrane oxygenation
ACT: Activated clotting time
CVP: Central venous pressure
MAP: Mean arterial pressure
HR: Heart rate
RR: Respiratory rate
PaO2: Partial pressures of oxygen
PaCO2: Partial pressures of carbon dioxide
PH: Potential of hydrogen
BMI: Body mass index
CABG: coronary arteries bypass grafting
